# Electronic Properties of DNA-Based Schottky Barrier Diodes in Response to Alpha Particles

**DOI:** 10.3390/s150511836

**Published:** 2015-05-21

**Authors:** Hassan Maktuff Jaber Al-Ta’ii, Vengadesh Periasamy, Yusoff Mohd Amin

**Affiliations:** 1 Low Dimensional Materials Research Centre (LDMRC), Department of Physics, University of Malaya, 50603 Kuala Lumpur, Malaysia; 2 Department of Physics, University of AL-Muthanna, 66001 AL-Muthanna, Iraq; 3 Department of Physics, Faculty of Science, University of Malaya, 50603 Kuala Lumpur, Malaysia; E-Mail: yusoffmohdamin@um.edu.my

**Keywords:** alpha particle, DNA, schottky diode, barrier height, richardson constant, series resistance

## Abstract

Detection of nuclear radiation such as alpha particles has become an important field of research in recent history due to nuclear threats and accidents. In this context; deoxyribonucleic acid (DNA) acting as an organic semiconducting material could be utilized in a metal/semiconductor Schottky junction for detecting alpha particles. In this work we demonstrate for the first time the effect of alpha irradiation on an Al/DNA/p-Si/Al Schottky diode by investigating its current-voltage characteristics. The diodes were exposed for different periods (0–20 min) of irradiation. Various diode parameters such as ideality factor, barrier height, series resistance, Richardson constant and saturation current were then determined using conventional, Cheung and Cheung’s and Norde methods. Generally, ideality factor or n values were observed to be greater than unity, which indicates the influence of some other current transport mechanism besides thermionic processes. Results indicated ideality factor variation between 9.97 and 9.57 for irradiation times between the ranges 0 to 20 min. Increase in the series resistance with increase in irradiation time was also observed when calculated using conventional and Cheung and Cheung’s methods. These responses demonstrate that changes in the electrical characteristics of the metal-semiconductor-metal diode could be further utilized as sensing elements to detect alpha particles.

## 1. Introduction

Organic semiconductor materials such as deoxyribonucleic acid (DNA) have taken center stage in biophysics, chemistry and biomedical research during the past few decades [1]. These types of materials present many advantages when applied to electronics and they enable rapid processing features for design and fabrication processes. For example, DNA has been studied extensively for its utilization and application in semiconductor devices, such as diodes and photovoltaic devices [2,3].

In the electronics industry, one common type of rectifying contact is the metal–semiconductor (MS) or Schottky diode. These diodes are widely utilized for application in radar technology, plasma diagnostics and telecommunications [4]. However, this type of diode has a disadvantage due to high bias voltage when used in mixed inorganic/organic-based diode setups [5]. The comparatively high essential bias voltage to operate mixed diodes imparts low sensitivities with low speeds and increasing noise from the voltage source. To overcome these problems, insertion of molecular layers between organic/inorganic Schottky diodes leads to new degrees of freedom for the mechanisms of essential device factors [6]. Recently, Schottky diodes were developed using thin DNA layers between the semiconductor and a top electrode [5], such as in the Al/DNA/p-Si structure. Applied as a temperature sensor, the diodes utilized the current transport characteristics between the temperature range of 200–300 K [7]. Gold-DNA-gold structures have also been fabricated to observe and measure optimized DNA responses to magnetic fields [8,9], radiation [10] and other phenomena [11].

Radiation effects play an important role in the electronic properties of the MS contacts, which were extensively studied during the last few decades. Lattice defect damages along the path of the radiation degrade the diode as a result of the defects acting as recombination centers. Recombination occurs when conduction electrons relax back to the valence band [12,13]. Lattice defects can also be caused by carrier trapping center, as discussed elsewhere [12,14,15].

In our present study conducted to reveal the effect of alpha radiation on DNA, we used mushroom-based DNA films deposited onto p-type Si wafers to fabricate Al/DNA/p-Si/Al junctions. Current-voltage (I-V) measurements were then performed to analyze the various electrical properties of the fabricated DNA-based diode. To the best of our knowledge, no such studies on similar structures have ever been reported before. The aim of this study is therefore to fabricate a DNA-based MS structure as a radiation sensitive material for potential utilization in alpha particle detectors/sensors. Understanding radiation-induced DNA damage may also enable interrogation into DNA damage and possible repair mechanisms, with obvious benefits in the biomedical field.

## 2. Methodology

### 2.1. Preparation of DNA Solution

Mushroom-based DNA was extracted from fresh fruiting bodies and used for Polymerase Chain Reaction (PCR) amplification. Collection of minute quantities of mycelium (0.1–1.0 gm) from the fruiting body (stipe) of oyster mushroom species (*Pleurotus* spp) was accomplished using sterilized tweezers. Standard procedures according to [16] were further employed to yield pure DNA samples prior to the PCR process. The DNA of all samples was amplified by PCR (PTC-100TM, MJ Research Inc., Ramsey, MJ, USA) using universal primers ITS1 forward (5′-TCC GTA GGTGA AC CTGCGG-3′) and ITS4 reverse (5′-TCCTCCGCTT ATT GATATGC-3′). Amplification reactions were performed in a total volume of 50 μL containing 10× PCR buffer 4 μL, dNTP mix 2.5 μL, 2.5 μL of each primer, 1 μL of Taq polymerase (Cosmo, Seongnam-si, Gyeonggi-do, Korea), 4 μL of genomic template DNA, and 26 μL of sterilized distilled water. PCR amplification was carried out in 30 cycles at 94 °C for 30 min and denatured at 50 °C for 60 min followed by annealing at 72 °C for an extension of 1 min. Initial denaturing at 95 °C was extended to 5 min and the final extension was at 72 °C for 5 min [17,18]. The concentration of the DNA was measured at 1.84 ng/µL (Nanodrop 2000, Thermo Scientific, Wilmington, DE, USA).

### 2.2. Preparation of Al/DNA/p-Si/Al Junctions

Junctions were prepared using a polished *p*-type Si wafer with [100] orientation with thickness and resistivity of 725–850 µm and 0.008–0.03 Ω·cm, respectively (Polishing Corporation of America, Polishing, Santa Clara, CA, USA). The RCA cleaning method was used to clean the wafer chemically; *i.e.*, 10 min boiling in NH_4_ + 6H_2_O + H_2_O_2_ followed by a 10 min boil in HCl + H_2_O_2_ + 6H_2_O solution. Then, Al metal was used as the back connection (sputtered, annealed at 570 °C for 3 min under N_2_ atmosphere) on the Si wafer p-type to obtain a low resistivity ohmic contact. To remove the native oxide layer on the surface of the Si wafer, immersion in HF/H_2_O (1:10) solution before rinsing in deionized water (18.2 MΩ·cm, Nanopure II water system, Barnstead Nanopure II water system, LakeBalboa, CA, USA) for 30 s were carried-out. Other necessary chemicals (NH_3_, H_2_O_2_, HF, HCl and acetone) from Sigma Aldrich (St. Louis, MO, USA) were used without further purification. After which, formation of the organic DNA layer was carried-out by using a micro syringe (Hamilton, MA, USA) containing 10.0 μL DNA with concentration of 1.80 ng/µL from the pre-prepared DNA stock solution. Schottky metal contacts were then deposited on the organic layer using a metal shadow mask by evaporating Al metal wire (Kurt J. Lesker, Hudson Valley, NY, USA) of 99.999% purity. The Al contacts had dimensions of 2.0 mm, 2000 Å and 3.14 × 10^−2^ cm^2^ of diameter, thickness and area, respectively. All evaporation processes were carried-out in a vacuum thermal metal evaporator-coating unit (Edward Auto 306, West Sussex, UK) pressurized to about 10^−7^ mbar.

**Figure 1:**
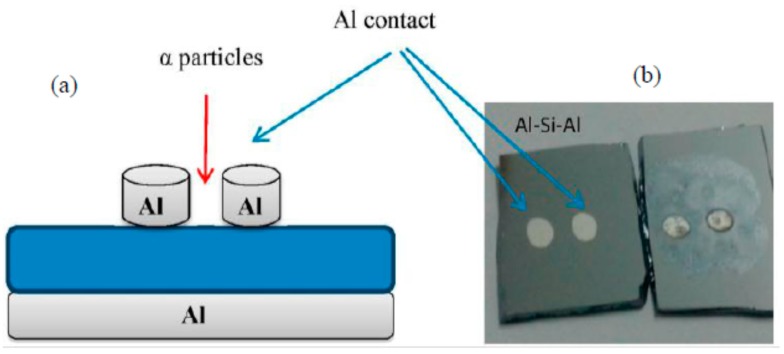
(**a**) Cross sectional and (**b**) top view of two Al/DNA/Si/Al surface-type Schottky diodes fabricated for further electrical characterization.

The prepared DNA-based devices were air-dried for 24 h in a 1 K clean room prior to irradiation of alpha particles using ^241^Am with an activity of 150 nCurie and t_1/2_ of 457 years for periods of 2, 4, 6, 8, 10, 20 min and again after 24 h. Its’ corresponding I-V profiles were also recorded in the dark using a Keithley SMU 236 electrometer (SMU-236, Keithley, Cleveland, OH, USA) at room temperature. Figure 1 depicts a schematic diagram of the DNA-based sensors fabricated in this work.

## 3. Results and Discussions

**Figure 2:**
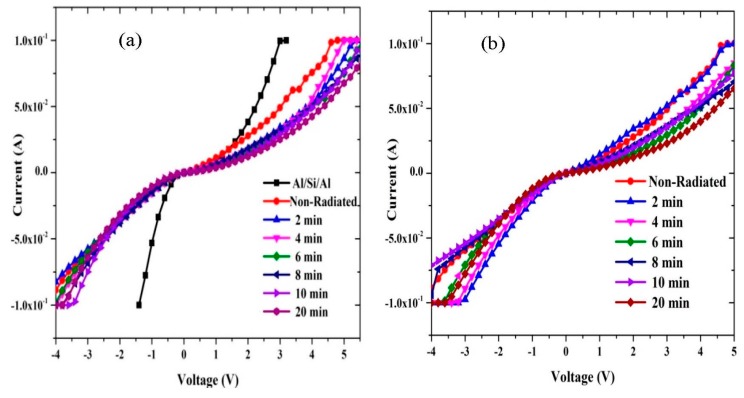
Relationship between I and V for forward and reverse biases at (**a**) 2, 4, 6, 8, 10 and 20 min and (**b**) measured after 24 h.

The reverse and forward bias I-V features of the Al/DNA/p-Si/Al and Al/Si/Al contact at room temperature are given in Figure 2 together with the former junction’s response after 24 h. As apparent from the figure, the I-V properties demonstrate a rectifying behavior. According to the thermionic emission theory, the I-V features of a junction follows the equations below [19];

(1) $$I=I_{o}\exp(\frac{qV}{nKT})\left[1-\exp(\frac{-qV}{KT})\right]$$

where:

(2) $${\text{I}}_{\text{o}}={\text{AA}}^{*}{\text{T}}^{2}\left(\frac{-\text{qΦ}}{\text{KT}}\right)$$

where the electron charge is q, the applied voltage is represented by V, the effective Richardson constant is A^*^ and equal to 32 A/cm^2^·K^2^ for p-type Si [20,21], A is the effective diode area, T is the absolute temperature, K is the Boltzmann constant, n is the ideality factor and Φ_bo_ is the zero bias barrier height. For values of V more than 3 kT/q, the ideality factor from Equation (1) can be re-written as:

(3) $$n=\frac{q}{KT}\left(\frac{dV}{d\ln I}\right)$$

**Figure 3:**
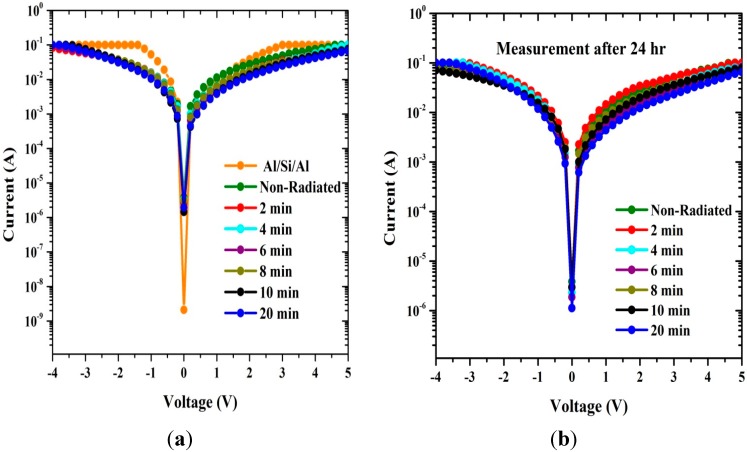
I-V characteristics of the Al/DNA/p-Si Schottky diode at room temperature. (**a**) before and after (**b**) 24 h.

The ideality factor can be determined from the slope of the linear region of the forward bias ln(I-V) characteristic, which is a measurement of the conformity of the junction to pure thermionic emission [22]. The n values for the samples Al/DNA/p-Si/Al Schottky diode was calculated using Equation (3). From the linear zone of the forward bias (Figure 3), for before and after irradiation, the I-V graph indicates that the affected series resistance is not significant. Value of the barrier height of Al/DNA/p-Si/Al in the two cases and Al/Si/Al Schottky junction was 0.4780 and 0.5078 eV, respectively. The values for the former junction are shown in Table 1, together with the ones measured after 24 h.

The barrier height was calculated based on the y-axis intercepts of the semi log-forward bias I-V plots using Equation (4). It should be noted that the barrier height is related to the potential barrier at the interface between the inorganic and organic layers, *i.e.*, at the DNA/p-Si interface:

(4) $$\text{Φ}=\frac{KT}{q}\ln\left(\frac{{AA}^{*}T^{2}}{I_{o}}\right)$$

**Table 1:** Values of ideality factor, barrier height and series resistance.

Radiation Time (min)	Al/DNA/Si/Al and Al/Si/Al										
	Conventional Method			Cheung-Cheung Method					Norde Method		
	n	Φ (eV)	R_S_ (Ω )	Φ (eV)	R_S_ (Ω )	n	R_S_ (Ω )	F(V)(V)		Φ (eV)	R_S_ (Ω )
Al/Si/Al	7.7478	0.5078	30.12	0.4259	41	1.5372	19.4057	0.497		0.3634	15.3107
0	9.9719	0.4780	46.304	0.4914	35	3.0217	20	0.47729		0.3632	15.4018
2	9.7934	0.5041	54.31	0.5208	42.	2.3643	24	0.495		0.3654	14.1393
4	10.5587	0.5004	62.81	0.5304	49	2.8682	27	0.4959		0.3685	12.5607
6	9.7301	0.5026	58	0.5241	46	1.5891	25	0.4984		0.3598	17.6020
8	9.7510	0.4982	57.38	0.5538	43	4.6512	25	0.5105		0.3653	14.2170
10	9.1727	0.5190	58.56	0.5560	43	3.5271	25	0.511		0.3493	26.3337
20	9.5912	0.5063	63.69	0.5526	48	4.6512	26	0.51		0.3503	25.3676

**Figure 4:**
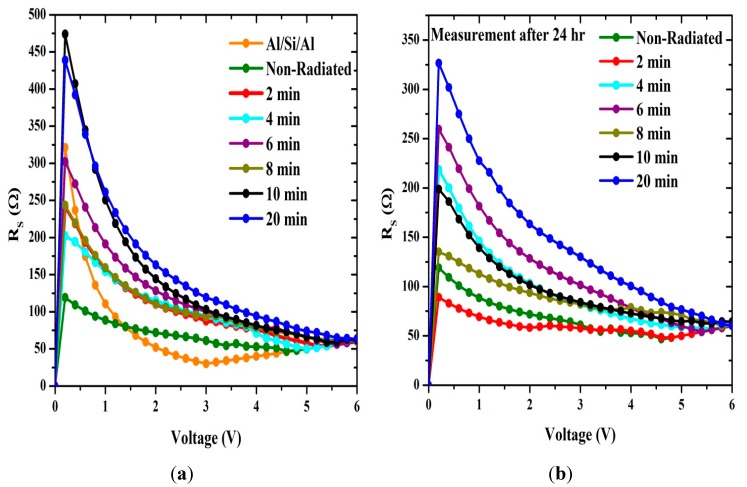
The relation between the series resistance and voltage generated using conventional method (**a**) before and (**b**) after 24 h.

The values of series resistances were calculated from the junction resistance (R_S_ = ∂V/∂I) obtained from the I-V properties of the diode. Resistance, R_S_ *vs.* voltage on the surface-type (Al/DNA/Si/Al) Schottky diode is shown in Figure 4. From the figure, it can be concluded that at low voltages (≤1.5 V), R_S_ values were the lowest except for the non-radiated samples, radiated for 10 and 20 min and for the Al/Si/Al junction. For irradiation periods beyond 24 h, the lowest values occur before radiation and at 2 and 8 min. However, above 2.0 V, the R_S_ value becomes insignificant for the latter sample. The highest R_S_ values occur in the sample radiated for 20 min followed by samples irradiated for 10 and 6 min. In the cases for after 24 h, the same trend was observed for samples radiated for 20, 6 and 4 min. This may refer to the phenomenon of DNA protecting itself by increasing its’ series resistance [23,24].

At high currents, there is always a deflection of the duality that relies on bulk series resistance and the interface state density [25,26]. The lower the series resistance and the interface state density, the better is the range over which ln I(V) yield a straight line. The Schottky diode factors such as the ideality factor, n, the series resistance, R_S_ and barrier height, Φ_bo_ were also measured using the technique developed by Cheung and Cheung’s [27]. Cheung and Cheung’s functions can be written as:

(5) $$\frac{dV}{d(\ln I)}={IR}_{S}+n\frac{KT}{q}$$

(6) $$H(I)=V-(\frac{KT}{q})\ln\left(\frac{I}{{AA}^{*}T^{2}}\right)$$

therefore:

(7) $$H(I)={IR}_{S}+{n\text{Φ}}_{b}$$

**Figure 5:**
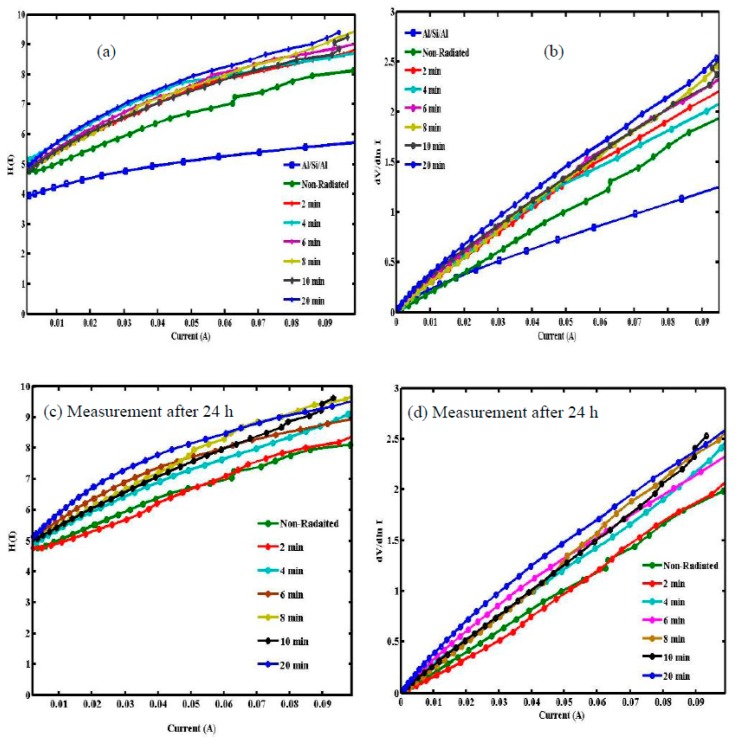
H(I) and dV/dln I *vs.* I obtained from forward bias I-V characteristics of Al/DNA/Si/Al Schottky junction diode diode (**a**,**b**) before and (**c**,**d**) after 24 h.

Figure 5 shows the experimental H(I) and dV/d(lnI) *vs.* I for the Al/DNA/Si and Schottky diodes at room temperature. H(I) *vs.* I (Figure 5a,c) meanwhile shows a straight line with the intercept at y-axis equal to nΦ. Φ was obtained by substituting the n value from Equation (5) and the data of the downward curvature region in the forward bias I-V features from Equation (7). The slope of this plot also limits R_S_, which can be used to check the accuracy of Cheung and Cheung’s method. From H(I) *vs.* I, the Φ and R_S_ values were measured and presented in Table 1 and Table 2. Equation (5) gives a straight line for the data of the downward curvature region in the forward bias I-V properties. Figure 5b,d show the plot of dV/d(lnI) *vs.* I, from which the values of n and R_S_ were calculated. As can be seen in the tables, the values of R_S_ obtained from both dV/d(lnI) *vs.* I and H(I) *vs.* I plots are in near agreement with each other. Radiation dose, however, does play an important role in changing series resistance values, thus the resistance increases gradually at low doses, self-protecting the DNA.

**Figure 6:**
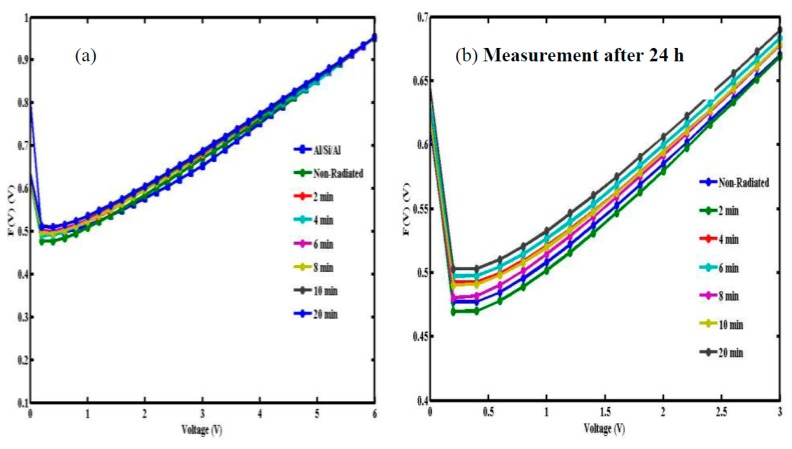
F(V) *vs.* voltage plots of non-radiated and radiated Al/DNA/Si Schottky diodes. diodes (**a**) before and (**b**) after 24 h.

The Norde method meanwhile is an alternative method to calculate the series resistance and barrier height [28,29,30]. The following function has been known in the modified Norde’s technique:

(8) $$F(V)=\frac{V}{\gamma }-\frac{KT}{q}\ln\left(\frac{I}{{AA}^{*}T^{2}}\right)$$

and active Schottky barrier height is given by:

(9) $$\text{Φ}=F(V_{\min})+\frac{V_{\min}}{\gamma }-\frac{KT}{q}$$

and:

(10) $$R_{S}=\frac{KT}{{qI}_{o}}$$

where F(V_min_) is the minimum point in the F(V) *vs.* V curve, V_min_ and I_o_ are the corresponding voltage and current, respectively. Graphs of F(V) *vs.* V for Al/DNA/Si before and after 24 h at room temperature is as displayed in Figure 6. From the plot F(V) *vs.* V, the values of Φ and R_S_ were determined (Table 1 and Table 2).

**Table 2:** Values of ideality factor, barrier height and series resistance measured after 24 h.

Radiation Time (min)	Al/DNA/Si/Al after One Day										
	Conventional Method			Cheung-Cheung Method					Norde Method		
	n	Φ (eV)	R_S_ (Ω )	Φ (eV)	R_S_ (Ω )	n	R_S_ (Ω)	F(V)(V)		Φ (eV)	R_S_ (Ω )
0	9.9719	0.4780	46.72	0.4713	38	3.3721	20	0.4772		0.3834	7.0697
2	10.1446	0.4736	48.41	0.4534	41	3.3333	22	0.4697		0.3308	11.5000
4	9.9168	0.4982	57.28	0.5042	43	1.3178	24	0.4927		0.3677	12.9375
6	10.1190	0.4982	54.11	0.5336	41	3.8760	23	0.4972		0.3030	33.5838
8	10.6707	0.4780	59.83	0.4779	52	1.3566	27	0.4805		0.3199	17.4831
10	9.7510	0.4982	62.52	0.4923	49	0.1899	26	0.4903		0.3100	25.6188
20	10.0429	0.5078	60.22	0.5477	47	5.0388	26	0.5031		0.2971	42.2497

**Figure 7:**
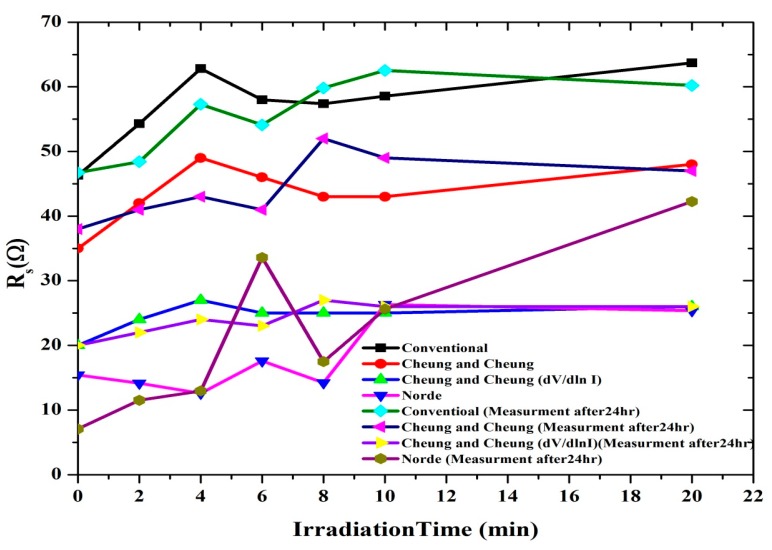
The relation between the series resistance and irradiation time.

According to the Norde method, the values of R_S_ were two orders of magnitude less when compared to the conventional method. From Figure 7, the series resistance was observed to have increased in all the methods with increasing irradiation time. This could be attributed to the increasing number of alpha particle tracks [31]. Also the increasing R_S_ of the material results in reduction in the reverse current as shown in Figure 2 and Figure 3, but series resistance measured using the conventional method remains the highest.

**Figure 8:**
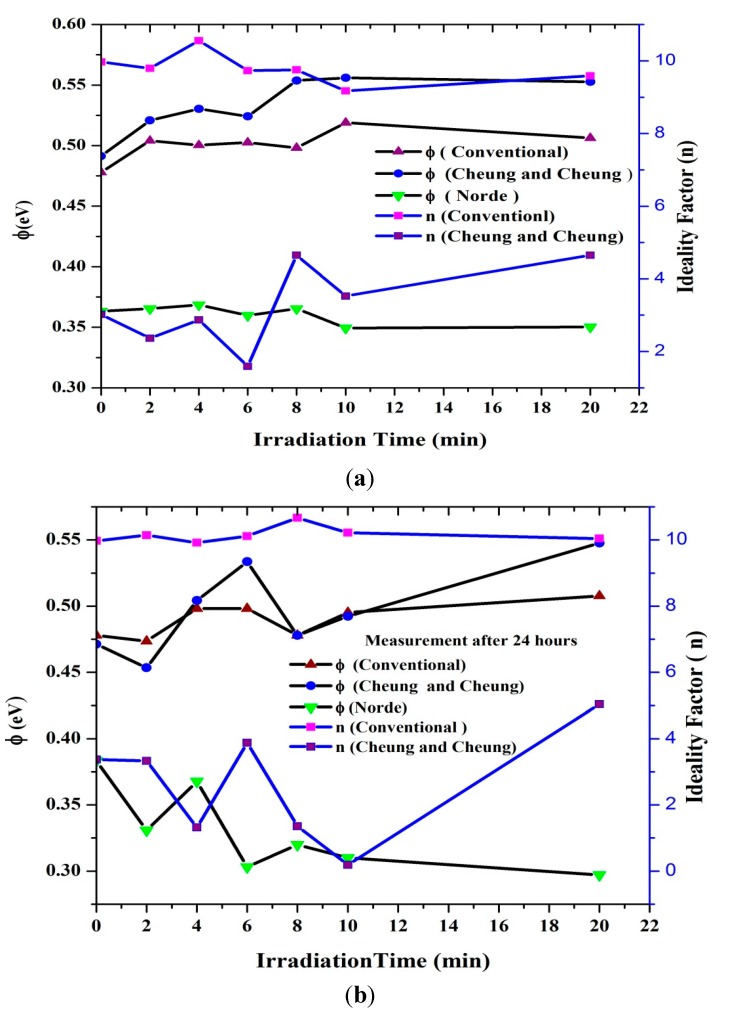
The relation between the ideality factor and barrier height with irradiation time. (**a**) before and (**b**) after 24 h.

Plots of Φ, n and R_S_ with radiation time are shown in Figure 7 and Figure 8, which clearly indicates the hypersensitivity phenomena of the DNA at low doses. Φ_b_ is the real barrier height taken from the low-voltage region of the forward I-V graph. The series resistance was obtained from the straight-line region observed in Figure 5. After which, the values of barrier heights and the series resistances were calculated from Equation (7) (Table 1 and Table 2). Results show that the values calculated are very close to each other. The values of n, Φ_b_ and R_S_ were obtained from conventional and Cheung-Cheung’s models**.** It indicates the values of n obtained from the dV/d(ln I) *vs.* I curve to be lower than the values obtained from the forward bias ln I *vs.* V plot. This can be attributed to the effect of the series resistance, interface states and voltage drop across the interfacial layer [32,33,34] and the irradiation effect [35].

Ideality factor equals one for an ideal diode, which means that the resulting current is only due to thermionic emission. In this work, the n values demonstrated greater than unity values when operated in the voltage range between −1 and +1 V [36]. When operated between −4 and +5 V, high values of n gives rise to a wide distribution of low barrier height Schottky diodes and interfacial thin layer [37]. This is due to an increase of defect density at the interface with irradiation or lateral inhomogeneous barrier height [38,39,40]. In this aspect, the effects of alpha particle with higher mass (4×) and charge (2×) compared to an electron, becomes greater than that of the electron and gamma rays (massless) [34].

At low doses, the ideality factor drops dramatically, which demonstrates the hypersensitivity phenomena of the DNA (Figure 8) and its self-protection. This phenomenon is similar to the behavior observed between survival curve and dosage [29,41,42]. Schottky barrier height on the other hand has an increased proportional relationship with the ideality factor as shown in Figure 8. From Figure 8b, barrier height values increases dramatically after 24 h of exposure as measured from Cheung and Cheung’s method and fluctuates according to conventional and Norde methods. This may arise due to the DNA oligonuctleotides ability to resist the alpha radiation as demonstrated by Figure 8a.

**Figure 9:**
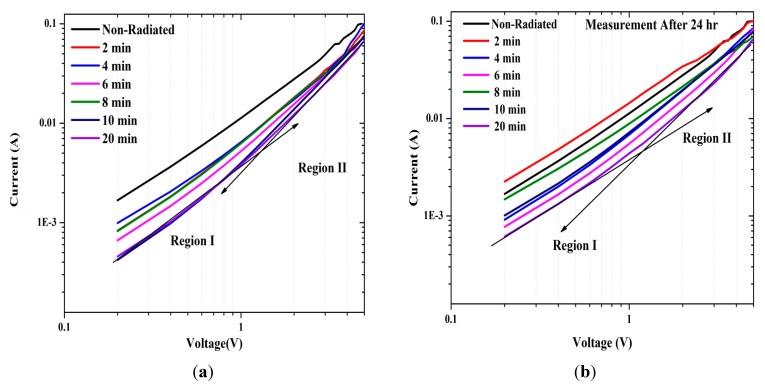
Double logarithmic plots of the Al/DNA/p-Si/Al junctions (**a**) before and (**b**) after 24 h.

Figure 9 displays the dual logarithmic plot of forward bias I-V properties of the Al/DNA/Si/Al junction. The log(I)-log(V) graphs clearly shows the power law behavior of the I-V curve. Space-charge-limited current (SCLC) affecting the diode and its charge transport can be shown through the I = V^m^ rule where m is the slope of each region, which corresponds to ohmic and SCLC. The m values of the region shown in Table 3, portrays two linear regions of the log(I)-log(V) plot of the forward bias I-V properties. The region (I) shows an ohmic region, while region (II) demonstrates the presence of the SCLC mechanism controlled by the traps.

**Table 3:** Values of m for regions (I) and (II) of the power law for Al/DNA/Si/Al and Al/DNA/Si/Al measured after 24 h.

Irradiation Time (min)	Al/DNA/Si/Al		Al/DNA/Si/Al after 24 h	
	Region (I)	Region (II)	Region (I)	Region (II)
0	1.16746	1.42133	1.1481	1.4343
2	1.38448	1.71641	1.22538	1.27533
4	1.35663	1.84725	1.33968	1.5977
6	1.38282	1.68783	1.35636	1.74065
8	1.31927	1.59468	1.18727	1.28848
10	1.47493	1.82395	1.3137	1.48376
20	1.3539	1.85096	1.15171	1.77029

**Figure 10:**
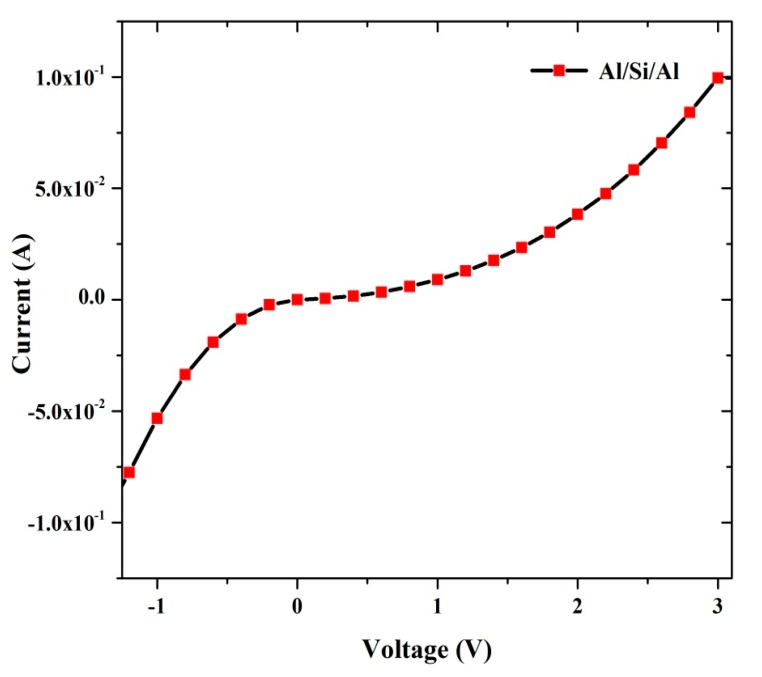
I-V curve of the Al/Si/Al junction in the absence of the DNA molecule.

**Figure 11:**
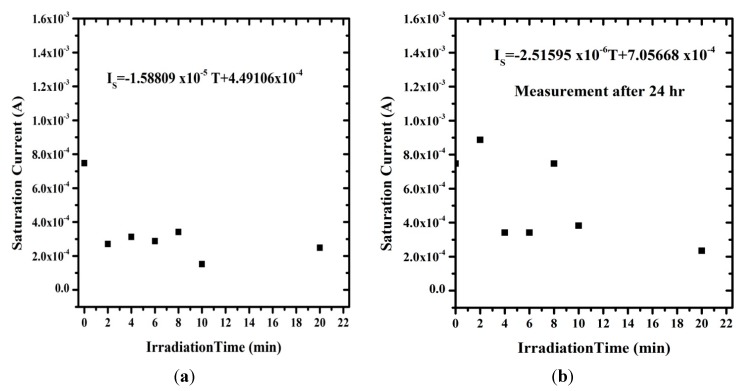
The relation between the saturation current and irradiation time (**a**) before and (**b**) after 24 h.

Figure 10 shows the I-V curve for the contact of Al/Si/Al sandwich structure in the absence of DNA, which generates a resistance of about 22.5 Ω and radiation effect induced current of about 10^−1^ A. This means that the radiation does not have any effect on the sample and the diode behaves as a good rectifier. Figure 11 shows the relation between saturation current under direct irradiation and after 24 h. In the first case, the saturation current was clearly lower than the non-radiated ones. However after 24 h, some of these irradiated samples generated higher currents. This observation of a decline in saturation current can be attributed to the rise of carrier resistance and potential barrier [43].

**Figure 12:**
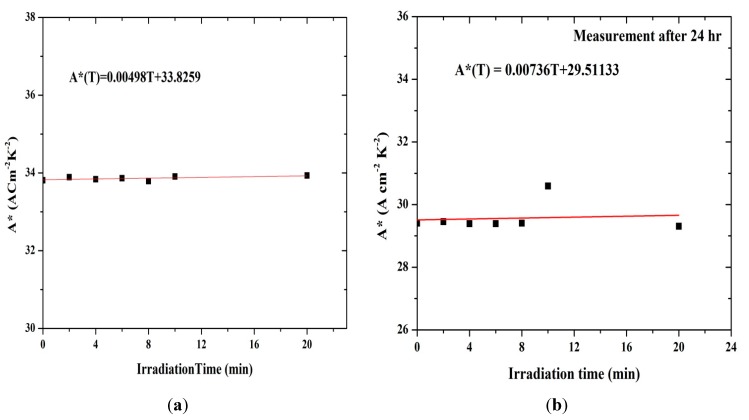
Irradiation time dependent Richardson constant for the MDM design (**a**) before and (**b**) after 24 h.

Figure 12 demonstrates that the Richardson constant is very sensitive to the radiation effect. The Richardson constant was measured from the I-V curve, and it increases with irradiation time. The ionizing radiation process leads to energy sedimentation in the metal, appearing as thermal heat and changing the material properties [14]. Work function of the metal/semiconductor junction changes, which provides sufficient energy for the charge carriers to get over the binding potential. Increasing number of alpha particles tracks also leads to increase in the number of holes thereby increasing the effective mass, which causes a lower rate of carriers to break through the potential barrier, reducing the current.

Due to the excitation of the material by ionizing radiation, such as by the alpha particles, a huge number of excited atoms are produced along its path, thereby increasing the number of electrons and R_S_. The number of electrons also decreases through the collision between the MSM electrodes. This results in increase in the barrier heights as in Table 4, followed by a decline in the current.

**Table 4:** Barrier height (ɸ) and Richardson constant (A*) against irradiation time in Al/DNA/Si/Al structures.

Irradiation Time (min)	ɸ (eV)	A*(ACm^−2^K^−2^)	ɸ (eV) (After 24 h)	A* (ACm^−2^K^−2^) (After 24 h)
0	0.4816	33.80976	0.4780	29.40648
2	0.5078	33.88901	0.4736	29.45637
4	0.5041	33.83585	0.4982	29.38977
6	0.5063	33.86438	0.4982	29.38978
8	0.5018	33.79057	0.4780	29.40648
10	0.5228	33.90649	0.4982	30.59743
20	0.5101	33.93433	0.5078	29.30825

## 4. Conclusions

We studied the I-V characteristics of Al/DNA/p-Si/Al Schottky diodes when radiated with alpha particles for various times (0, 2, 4, 6, 8, 10 and 20 min). Influence of the particles was also studied after 24 h. Various diode parameters such as ideality factor, barrier height, Richardson constant, series resistance and saturation current were determined from the I-V features at different irradiation times using conventional, Cheung and Cheung’s and Norde methods. We have shown, from this study, that the alpha particle effect can be demonstrated by the electrical characterization carried-out. Using the conventional technique, series resistance increased from 46.0 to 62.0 Ω between the radiation time of 0–20 min and after 24 h between 46.0 to 60.0 Ω for the same time periods (Figure 4 and Table 1 and Table 2). Barrier height values were observed to generally increase after 2, 4, 6, 8, 10 and 20 min of radiation and 4 to 20 min after 24 h. Irreversible changes to the structure properties can be observed, but it was also observed that at 4 min, reversible properties back to non-radiated ideality factor values were observed. These may indicate a self-protecting and self-repairing phenomenon of the DNA against the radiation or the hypersensitivity phenomena. The Richardson constant was also affected: initially increasing but followed by a reduction after 24 h. The overall results of this investigation show that the Al/DNA/Si structure could be potentially employed as a radiation sensor as the effect of radiation was clearly quantified by measurement of the various electronic parameters.
